# Development of aggregated state chemistry accelerated by aggregation-induced emission

**DOI:** 10.1093/nsr/nwaa199

**Published:** 2020-08-31

**Authors:** Yujun Xie, Zhen Li

**Affiliations:** Institute of Molecular Aggregation Science, Tianjin University, China; Institute of Molecular Aggregation Science, Tianjin University, China; Joint School of National University of Singapore and Tianjin University, International Campus of Tianjin University, China; Department of Chemistry, Wuhan University, China

## Abstract

The foundation of aggregation-induced emission (AIE) has greatly affected the design strategies of luminous materials, and accelerated the relevant theoretical and practical application investigation. The AIE concept also inspires the exploration on “aggregated state chemistry” and enlightens new research areas of room-temperature phosphorescence and mechanoluminescence.

Light is an essential ingredient of human civilization, and has been tightly associated with society and everyone's daily life. Light can be emitted from various kinds of luminophores, and the concept of aggregation-induced emission (AIE) was a breakthrough in the field of organic light-emitting materials, which experienced stages of pursuing truth, discarding prejudices and creating new knowledge.

For many luminogens, concentration quenching is a well-known recognition method, which causes many difficulties encountered in the development of materials and devices related to strong emission in aggregated states. For example, the emission photos of a typical organic luminophore, i.e. fluorescein, in different mixture solvents (Fig. 1a) clearly demonstrate the process of aggregation-caused quenching (ACQ) [1]. To address or alleviate this undesirable drawback, numerous chemical and physical engineering methodologies have been applied, such as the introduction of a bulk steric group or its dispersion into the host matrix to prevent intermolecular aggregation [2]. However, these attempts realized limited success, as the aggregation is a natural process and cannot be totally blocked. Possibly influenced by these results, little attention has been paid to photophysical properties in the aggregates, and some occasional abnormal emission phenomena without ACQ in the literature have not attracted wide consideration, possibly due to the lack of a convincing theoretical explanation and systematic investigation. For example, in 1997, Steinhuber and coworkers reported that the introduction of electron-withdrawing substituents into the oligophenylenevinylenes would reduce the fluorescence yield (Φ_F_) in low-viscosity dilute solution [3], but increase it at high viscosities and condensation to solid phases. These phenomena were attributed to the suppression of nonradiative torsional deactivation and formation of *J*-aggregates with high radiative rate in the condensed phase. Thus, the prejudice has been embedded deeply in people's minds that the aggregation is detrimental to light emission.

**Figure 1.**
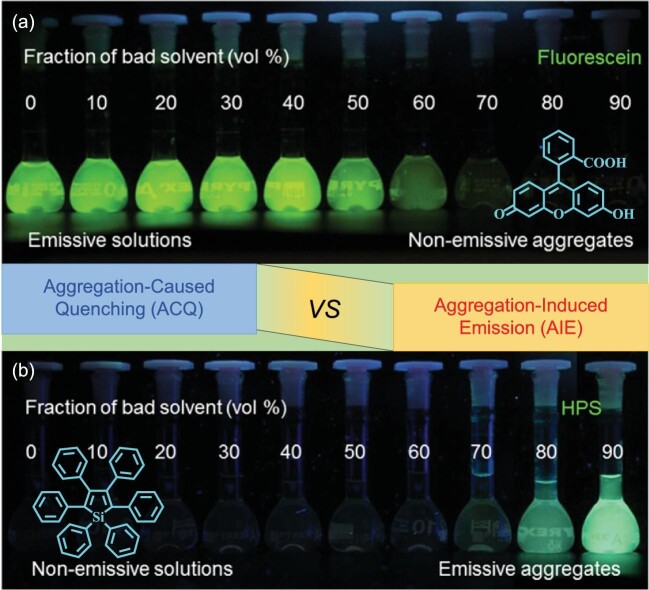
(a) Fluorescence photographs of fluorescein (15 μM) in water/acetone mixtures with different fractions of acetone. (b) HPS (20 μM) in THF/water mixtures with different fractions of water. Insets display the molecular structure (adapted with permission from ref. [1]).

A milestone was reached in 2001, when Tang's group observed an interesting phenomenon; that is, as shown in Fig. 1b, hexaphenylsilole (HPS) with a highly distorted and flexible molecular configuration was nonemissive in a good solvent of tetrahydrofuran (THF), but the emission was gradually turned on and increased accompanied by the addition of water as the poor solvent [4]. As aggregates were formed under high water fraction, it was found that aggregation could be utilized to enhance luminescence, i.e. AIE. The subsequent extensive theoretical and experiment analyses demonstrated that the distorted structure inhibited the compact intermolecular interactions in the aggregated state, while the low-frequency vibration or rotation mode was restrained. Therefore, the aggregates do not quench but intensify the emission because the nonradiative transition was greatly suppressed. Inspired by this phenomenon, thousands of AIE small molecules and polymers were reported, and among them HPS, tetraphenylethene (TPE) and 2^′^-butoxy-5^′^-phenyl-1,1^′^:3^′^,1^′^-terphenyl (TPP) (Fig. 2) were the famous units in designing AIE compounds. Meanwhile, several mechanisms have been proposed, such as restriction of intramolecular rotation, restriction of intramolecular vibration, restriction of twisted intramolecular charge transfer, vibration-induced emission, conformational planarization, *J*-aggregation formation, *E*/*Z* isomerization and excited-state intramolecular proton transfer. However, none of these was capable of describing all of the AIE situations perfectly, especially the disputes regarding the excited-state dynamic of TPE. Actually, combined with the luminous characteristic of AIEgens, the nonradiative transition rate was largely suppressed in the aggregated state, and thus the restriction of intramolecular motion should be a more comprehensive and accurate mechanism [5].

**Figure 2.**
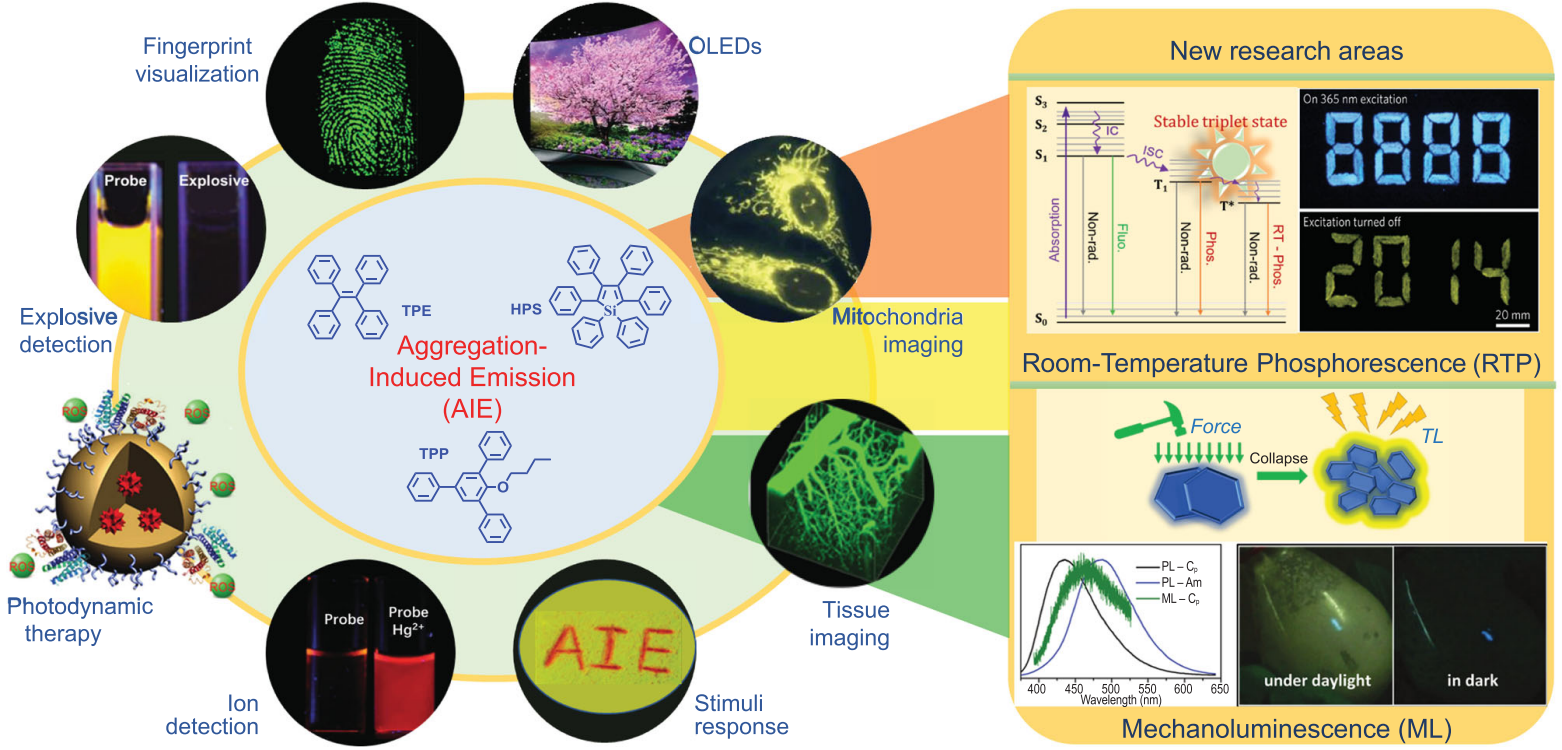
Left: typical AIE molecules of TPE, HPS and TPP and their technological applications. Right: schematic of new research areas derived from AIE, i.e. RTP and ML; insets show RTP images under ultraviolet excitation turned on/off and the ML spectra images taken under daylight and in dark conditions (adapted with permission from refs [2,8,9]).

The AIE concept deeply affects the design of luminous materials, and the new generation of luminogens has been applied and prompted a breakthrough in many fields (Fig. 2) [1,2]. For example, AIEgens are highly sensitive to the external stimuli because of the distorted structure, resulting in the mechanochromism effect in most AIE compounds; also, the full-color high-efficiency organic light-emitting diodes are developed because of the high luminous efficiency. In the fingerprint visualization and tissue and mitochondria imaging, AIEgens gather together selectively to the biological target, leading to bright and clear imaging without the drawback of photobleaching. In explosive and ion detection, the bright emission of aggregated AIEgens is sensitively responsive to the target analytes. Especially, the valuable work from Liu’s group has greatly prompted the application of AIEgens in the field of photodynamic therapy. More importantly, great effort should be made into the practical application in daily life and guard human lives, especially in this special period of early 2020, under the threat of COVID-19. We are pleased to notice that the precious quick detection kit with the technique of AIE dots has been developed quickly.

Furthermore, the AIE concept has enlightened and triggered the exploration of what happens in the aggregated state, and has enhanced the search for the correlation of photophysical properties with molecular structures and intermolecular packing style [5]. Whether it is an AIE compound or not, the intermolecular interactions are intensified but the molecular motion is suppressed upon aggregation, and luminous characteristics are difficult to predict. For example, Tang and coworkers further reported that nonluminescent compounds with nonaromatic or nonconjugate groups, such as sodium alginate [6], exhibit strong emissions in the aggregated state, completely beyond the predictions of traditional photochemical theory. In 2018, Li's group proposed the concept of ‘molecular uniting set identified characteristic (MUSIC)’ to emphasize the unique photophysical properties in the aggregated state from the perspective of molecular configuration and intermolecular packing style, welcoming the flourishing of the research area of ‘aggregated state chemistry’ [7], which focuses on the chemistry and physical properties of molecules in aggregates much different from those of their single molecular state.

Moreover, pure organic room-temperature phosphorescence (RTP) [8] and mechanoluminescence (ML) [9] (Fig. 2), which are derived from the research on solid-state emission behavior, have become hot topics with an emphasis on molecular packing. ML properties have been reported in many AIEgens owing to their high emission efficiency and intense intermolecular interaction as aggregates, while the RTP behavior was generally observed in the aggregated state and can be considered phosphorescence AIEgens [10]. As a new research area, although the organic RTP and ML are still in the initial stage, the concept of AIE has greatly prompted progress in devising luminescent materials and the relevant theoretical studies.

Through the great efforts of scientists, chemistry is focused on single molecules on one hand, but increasing attention is being devoted to molecular aggregates on the other. This trend has been gradually extended to related research fields. Although currently there are numerous thorny subjects in AIE, such as the complicated anti-Kasha phenomenon in the aggregated state, the low targeting capability of AIEgens in the nanoaggregate state, the far-red and near-infrared ray AIEgen platform and photoswitchable AIE building block, encouraging progress has been made consistently. However, with the further exploration of ‘aggregated state chemistry’, more novel aggregate phenomena, not only limited to the luminescence, remain to be discovered, with more exciting MUSICs.
